# Visual Sensing of *β*-Glucosidase From Intestinal Fungus in the Generation of Cytotoxic Icarisid II

**DOI:** 10.3389/fchem.2022.919624

**Published:** 2022-05-27

**Authors:** Gang Wang, Fei Yan, Yufei Wang, Yingping Liu, Jingnan Cui, Zhenlong Yu, Lei Feng, Tony D. James, Chao Wang, Ying Kong

**Affiliations:** ^1^ College of Basic Medical Sciences, College of Pharmacy, Academy of Integrative Medicine, Dalian Medical University, Dalian, China; ^2^ Second Affiliated Hospital of Dalian Medical University, Dalian, China; ^3^ State Key Laboratory of Fine Chemicals, Dalian University of Technology, Dalian, China; ^4^ Department of Chemistry, University of Bath, Bath, United Kingdom; ^5^ School of Chemistry and Chemical Engineering, Henan Normal University, Xinxiang, China

**Keywords:** β-glucosidase, fluorescent probe, Pichia terricola M2, iIcariside Ⅱ, endometrial cancer

## Abstract

*β*-Glucosidase (*β*-Glc) is an enzyme capable of the selective hydrolysis of the *β*-glycosidic bond of glycosides and glycans containing glucose. *β*-Glc expressed by intestinal microbiota has attracted increasing levels of interest, due to their important roles for the metabolism of exogenous substances in the gut. Using the 2-((6-hydroxy-2,3-dihydro-1*H*-xanthen-4-yl)methylene)malononitrile fluorophore (**DXM-OH**, λ_em_ 636 nm) and the recognition group *β*-Glucose, an enzymatic activatable turn-on fluorescent probe (**DXM-Glc**) was developed for the selective and sensitive sensing of *β*-Glc. In addition, **DXM-Glc** could be used to sense endogenous *β*-Glc in living fungal cells. Using **DXM-Glc**, *Pichia terricola* M2 was identified as a functional intestinal fungus with *β*-Glc expression. *P. terricola* M2 could transform the flavone glycoside Icariin to Icariside Ⅱ efficiently, which confirmed the metabolism of glycosides in the gut mediated by fungi. Furthermore, Icariside Ⅱ could inhibit the proliferation of human endometrial cancer cells (RL 95-2 and ishikawa) significantly, suggesting the metabolic activation of Icariin by intestinal fungi *in vivo*. Therefore, **DXM-Glc** as a probe for *β*-Glc provided a novel technique for the investigation of the metabolism of bioactive substances by intestinal microbiota.

## Introduction

*β*-Glucosidase (*β*-d-glucopyranoside glucohydrolase, *β*-Glc) [E.C.3.2.1.21] is a well-known functional enzyme for the hydrolysis of the glycosidic bond of carbohydrate moieties. As a biocatalyst, *β*-Glc was been widely used in industrial preparations, exhibiting the advantages of improved conversion rate, mild reaction conditions, while being environmental-friendly, and enabling the simple purification of products(Hong, et al., 2012; Hati, et al., 2015; Zhong, et al., 2016; Abdella, et al., 2018).

Moreover, gut bacteria (e.g. *Streptococcus thermophilus*, *L. acidophilus*, *L. delbrueckii*, *L. casei*, *L. plantarum*, *L. fermentum*) express *β*-Glc, and participate in the metabolism of glycosides and carbohydrates from foods and pharmaceuticals(Donkor et al., 2008; Shen, et al., 2013). The glycosides from plants or foods exhibit poor intestinal absorption, however, the corresponding aglycones which are the metabolites generated by intestinal microbiota are more permeable and bioactive(Rekha et al., 2011). The aglycone metabolites are the main forms transported across the epithelial membrane and are likely to be the active forms *in vivo*(Quan et al., 2012a; Quan et al., 2012b). Therefore, intestinal bacteria or fungi with active *β*-Glc play an important role in the absorption, and metabolic activation of glycosides and carbohydrates. As such, the characterization and further exploration of intestinal *β*-Glc requires more investigation.

On the basis of the special catalytic characteristics of *β*-Glc, several fluorescent carbon dots have been developed as fluorescent biosensors for the sensitive determination of Glucosidase, including *α*-Glc and *β*-Glc(Kalaiyarasan, et al., 2019; Kong, et al., 2020; Liu, et al., 2020; Wang, et al., 2020). While, enzymatic fluorescent probes have been widely used to sense biological enzymes, exhibiting the advantages of high sensitivity, high selectivity and facilitate *in vivo*/*in vitro* imaging(Feng, et al., 2021; Li, et al., 2020; Liu, et al., 2021; Ning, et al., 2019; Tian, et al., 2021; Yan, et al., 2022; Zhang, et al., 2021). Using the excited-state intramolecular proton transfer (ESIPT) phenomenon, flavonol derivatives have been used as fluorescent indicators for *β*-Glc activity(Reszka, et al., 2020). However, an enzymatic activatable long wavelength fluorescent probe is still desirable for the sensing and determination of intestinal *β*-Glc.

In this study, a turn-on fluorescent probe (**DXM-Glc**) has been developed determining *β*-Glc activity sensitively and selectively. When combined with cultures of intestinal fungi, visual identification of target fungus expressing *β*-Glc was successfully achieved. Furthermore, using the identified intestinal fungus, Icarisid Ⅱ was released as a metabolite of Icariin, which exhibited significant cytotoxicity toward endometrial cancer cells.

## Materials and Methods

### Materials and Apparatus

Chromatographic methanol for HPLC was purchased from sigma-aldrich (MERCK, United States). All of the chemical reagents and solvents for the synthesis were obtained from Tianjin Kemiou Chemical Reagent Co., Ltd (Tianjin, P.R. China). The agar, glucose, Na_2_HPO_4_, and NaH_2_PO_4_ were produced by Dalian Meilun Biotechnology Co., Ltd (China). *β*-Glucosidase (*β*-Glc), *α*-Glucosidase (*α*-Glc), Lipase, Human serum albumin (HSA), *β*-Galactosidase (GAL), and *β*-N-Acetylglucosaminidase (NAG) were obtained from sigma-aldrich (MERCK, United States). Recombinant human carboxylesterases (CES1b, CES1c, and CES2) were purchased from Corning Incorporated Life Sciences.

NMR spectra were measured using Bruker-600 with tetramethylsilane (TMS) as the internal standard (Bruker, United States). HRESIMS data were acquired on an AB SciexX500r TOF mass spectrometer (AB Science, United States). Fluorescence microscopic imaging was conducted using a Leica Confocal Microscope (Leica Microsystems, Germany). The bioassay solutions in 96-well plates were analyzed using a BioTek Synergy H1 microplate reader (BioTek, United States). HPLC-UV analysis was performed using a Waters e2695 (Waters, United States).

### Synthesis of Fluorescent Probe DXM-Glc

**DXM-Glc** was synthesized according to the route shown in Supplementary Scheme S1.

In general, to a solution of **DXM-OH** (55.2 mg, 0.20 mmol) in 10 ml of dry DCM, 2,3,4,6-tetra-*O*-acetyl-*α*-d-glucosyl bromide (164.4 mg, 0.4 mmol), Cs_2_CO_3_ (163 mg, 0.5 mmol) were added. The reaction mixture was stirred at room temperature overnight, filtered, and evaporated. The residue was dissolved in 10 ml of MeOH, and CH_3_ONa (108 mg, 2 mmol) was added. The mixture was stirred at room temperature for 1 h, neutralized with 1M HCl, filtered, and evaporated. The residue was purified by HPLC to afford 20.5 mg **DXM-Glc** as dark red powder. Yield: 23.4% in two steps. The chemical structure of **DXM-Glc** was determined by ^1^H, ^13^C NMR and HR-MS data (Supplementary Figures S1-S3).

^1^H NMR (600 MHz, DMSO-*d*_6_) *δ*_H_ 8.17 (s, 1H), 7.46 (d, *J* = 8.5 Hz, 1H), 7.36 (s, 1H), 7.25 (s, 1H), 6.96 (d, *J* = 7.1 Hz, 1H), 5.41 (d, *J* = 4.7 Hz, 1H), 5.15 (d, *J* = 4.0 Hz, 1H), 5.08 (d, *J* = 4.8 Hz, 1H), 5.04 (d, *J* = 7.5 Hz, 1H), 4.59 (s, 1H), 3.69 (d, *J* = 8.6 Hz, 1H), 3.49 (d, *J* = 5.7 Hz, 1H), 3.25 (d, *J* = 5.2 Hz, 1H), 3.19 (d, *J* = 4.8 Hz, 1H), 3.12 (s, 2H), 2.75 (s, 2H), 2.63 (s, 2H), 1.82–1.69 (m, 2H). ^13^C NMR (150 MHz, DMSO-*d*_6_) *δ*_C_ 160.22, 159.51, 153.74, 150.53, 132.39, 128.88, 126.82, 118.07, 116.63, 116.11, 114.61, 109.94, 103.66, 100.39, 77.55, 76.96, 73.61, 69.98, 68.11, 61.04, 28.57, 24.80, 20.44. HRMS cacld for [M + H]^+^ 439.1500, found *m/z* 439.1508.

### Hydrolysis of DXM-Glc Catalyzed by *β*-Glucosidase

In a phosphate buffer solution (pH 7.4, 100 mM), *β*-Glc (100 μg/ml) and **DXM-Glc** (10 μM, DMSO <1%, *v/v*) were co-incubated at 37°C for 30 min. Then, dimethyl sulfoxide (33%, *v/v*) was added to inactivate the *β*-Glc activity and terminate the enzymatic reaction. When the denatured proteins were precipitated by centrifugation at 20,000 ×*g* for 20 min, the fluorescence intensity corresponding to the production of **DXM-OH** was measured using a Microplate reader with an excitation wavelength of 600 nm and emission wavelength of 636 nm.

The fluorescence response of **DXM-Glc** (10 μM) toward *β*-Glc with different concentrations (0, 2, 5, 10, 20, 40, 60, 80, 100 μg/ml) has been recorded using an excitation laser at 600 nm. The linear relationship between the fluorescence intensity and *β*-Glc concentrations was calculated using linear regression equation. The limit of detection (LOD) was calculated using 3*σ*/slope.

### Interference by Various Species on the Fluorescence Emission of DXM-Glc

Various ions including K^+^, Na^+^, Mg^2+^, Ca^2+^, Ba^2+^, Zn^2+^, Cu^2+^, Ni^2+^, Sn^2+^, Mn^2+^, CO_3_^2-^, SO_4_^2-^ and amino acids (200 μM) were co-incubated with **DXM-Glc** in phosphate buffer at 37°C for 30 min, respectively. Then, the fluorescence intensity was measured using a Microplate reader (λ_ex_ 600 nm/λ_em_ 636 nm).

The selectivity of **DXM-Glc** toward *β*-Glc was evaluated in the presence of other biological enzymes *β*-Glc, *α*-Glc, HSA, GAL, NAG, CES1b, CES1c, and CES2 (100 μg/ml). The fluorescence responses of **DXM-Glc** toward these enzymes were measured using Microplate reader (λ_ex_ 600 nm/λ_em_ 636 nm).

### Visual Sensing of *β*-Glc With Intestinal Fungus

The fungi strains were cultured in martin broth modified (MTB: glucose 200 g/L, yeast extract 2 g/L, KH_2_PO_4_ 1 g/L, Mg_2_SO_4_ 0.5 g/L) at 32°C, 160 r/min. 36 h later, the probe **DXM-Glc** was added with a final concentration of 20 μM and incubated for 4 h. Then, the fluorescence intensity was measured using Microplate reader (λ_ex_ 600 nm/λ_em_ 636 nm). Similarly, after incubation, *Pichia terricola* M2 cells were washed with PBS and diluted in saline. The suspensions were dropped on glass slides and imaged using a laser confocal microscope (λ_ex_ 561/λ_em_ 600–660 nm).

### Biotransformation of Icariin by *Pichia terricola* M2

*Pichia terricola* M2 was cultured in MTB medium at 32°C, 160 r/min. Icariin was added with a final concentration of 10 mg/ml. Five days later, the fungal cells were collected from the fermentation broth by centrifugation (5,000 *g*, 10 min). Then, the supernatant was inactivated by acetonitrile for HPLC analysis, which was used to confirm the production of Icariside II. Icariside II was purified by extraction of the fermentation broth using ethyl acetate, and the metabolite was then purified using preparative HPLC.

Icariin, yellow powder. ^1^H-NMR (DMSO-*d*_6_, 600 MHz) *δ*_H_ 12.57 (1H, s), 7.89 (2H, d, *J* = 9.0 Hz), 7.13 (2H, d, *J* = 9.0 Hz), 6.63 (1H, s), 5.35 (1H, s), 5.28 (1H, s), 5.16 (1H, t, *J* = 7.2 Hz), 5.12 (1H, s), 5.05 (1H, d, *J* = 4.2 Hz), 5.00 (1H, d, *J* = 7.2 Hz), 4.98 (1H, d, *J* = 4.8 Hz), 4.73 (1H, d, *J* = 3.6 Hz), 4.66 (1H, d, *J* = 3.6 Hz), 4.62 (1H, t, *J* = 3.6 Hz), 4.00 (1H, s), 3.85 (3H, s), 3.71 (1H, m), 3.57 (1H, dd, *J* = 14.4, 6.6 Hz), 3.46 (4H, m), 3.30 (4H, m), 3.14 (2H, m), 3.08 (1H, m), 1.69 (3H, s), 1.60 (3H, s), 0.79 (3H, d, *J* = 6.6 Hz). ^13^C-NMR (DMSO-*d*_6_, 150 MHz) *δ*_C_ 178.30, 161.42, 160.52, 159.09, 157.33, 153.02, 134.65, 131.12, 130.57, 122.27, 122.14, 114.09, 108.30, 105.60, 101.99, 100.54, 98.13, 77.19, 76.61, 73.36, 71.11, 70.71, 70.31, 70.08, 69.66, 60.63, 55.51, 25.47, 21.42, 17.87, 17.46. HR-MS: *m/z* 677.2430, [M + H]^+^, calcd for C_33_H_41_O_15_, 677.2440 (Supplementary Figures S4-S6).

Icariside II, yellow powder. ^1^H-NMR (DMSO-*d*_6_, 600 MHz) *δ*_H_ 12.52 (1H, s), 10.85 (1H, s), 7.85 (2H, d, *J* = 9.0 Hz), 7.11 (2H, d, *J* = 9.0 Hz), 6.31 (1H, s), 5.26 (1H, s), 5.15 (1H, t, *J* = 6.6 Hz), 4.97 (1H, d, *J* = 4.2 Hz), 4.71 (1H, d, *J* = 4.2 Hz), 4.64 (1H, d, *J* = 5.4 Hz), 3.98 (1H, br s), 3.85 (3H, s), 3.47 (1H, m), 3.41 (1H, dd, *J* = 14.4, 6.6 Hz), 3.33 (1H, m), 3.13 (1H, m), 3.06 (1H, m), 1.67 (3H, s), 1.02 (3H, s), 0.78 (3H, d, *J* = 6.0 Hz). ^13^C-NMR (DMSO-*d*_6_, 150 MHz) *δ*_C_ 177.99, 161.68, 161.28, 158.85, 156.75, 153.79, 134.43, 131.03, 130.42, 122.41, 122.27, 114.06, 105.95, 104.17, 101.96, 98.35, 71.11, 70.65, 70.30, 70.07, 55.49, 25.43, 21.17, 17.79, 17.46. HR-MS: *m/z* 515.1909, [M + H]^+^, calcd for C_27_H_31_O_10_, 515.1912 (Supplementary Figures S7-S9).

### Inhibitory Effect of Icariside II on Human Endometrial *Cancer* Cells

First, serum-free medium was added to the real-time label free cell analyzer to remove the background value, and cells (3 ×10^4^) were inoculated into a 16-well culture plate of the cell analyzer. Then, a certain concentration of Icariin II was added respectively (0, 10, 20, 40 μM). After mixing, the sample was placed in the real-time unmarked cell analyzer, and the cell proliferation curve was obtained after 48 h.

Cell viability was determined using a CCK-8 assay. In brief, 3 × 10^3^ cells were seeded into 96-well culture plates allowed to adhere overnight, and then the cells were changed to fresh medium containing various concentrations of Icariside II dissolved in DMSO (final concentration, 0.1%). After incubation for 24, 48, and 72 h, CCK-8 was added, and the absorbance was measured at 450 nm by EnSpire^®^ Multimode Plate Reade (Perkin Elmer, United States). Cell viability in the vehicle control groups was considered 100%. Each assay was carried out in triplicate.

To analyze the effects of Icariside II on colony formation, single cells (3 × 10^3^ per well) were seeded in 6-well plate containing 2 ml growth medium with 10% FBS and cultured for 24 h. Then, the medium was removed, and cells were treated with various concentrations of Icariside II (0, 10, 20, 40 μM). After 24 h, cells were washed with PBS and supplemented with fresh growth medium, cells were routinely incubated for about 10 days until colonies were large enough to be visualized. Then colonies were stained with 0.1% crystal violet and counted.

### Statistical Analysis

The measurements of fluorescence intensities and cell viabilities were repeated at least three times. Data are represented as the mean ± standard deviation (SD). Analysis of variance and Student’s t-test were used to compare the values of the test and control samples. *p* < 0.05 was the statistically significant difference. Graphpad Prism 8 software was used for all statistical analysis.

## Results and Discussion

### DXM-Glc as an Off-On Fluorescent Probe for *β*-Glc

**FIGURE 1:**
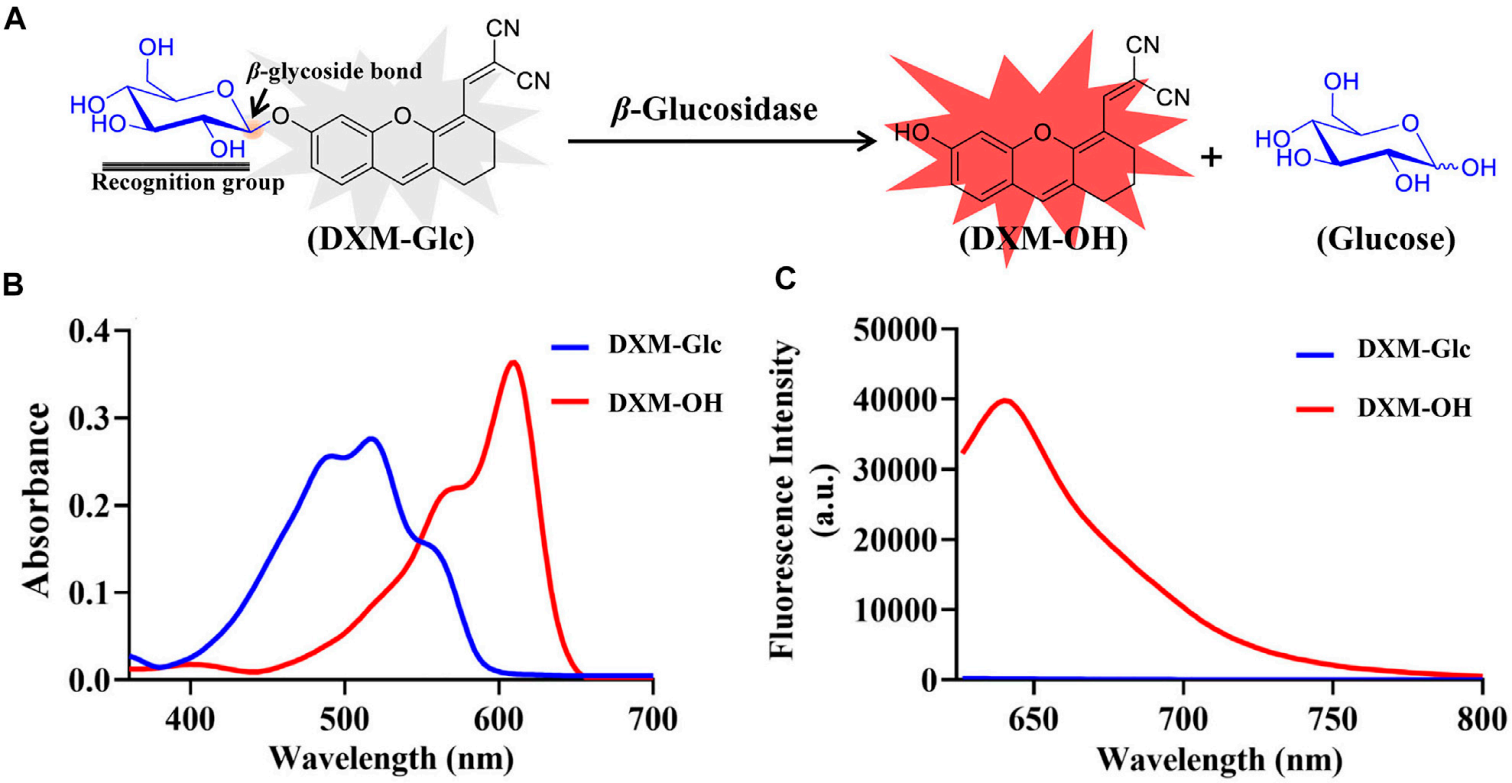
Design of fluorescent probe for *β*-Glc. **(A)** Illustration for the hydrolysis of **DXM-Glc** mediated by *β*-Glc. **(B)** Absorbance spectra of **DXM-Glc** and **DXM-OH**. **(C)** Fluorescence spectra of **DXM-Glc** and **DXM-OH** (λ_ex_ 600 nm).

*β*-Glc is known to mediate the cleavage of *β*-*O*-glycosidic bond of glycosides and glycans. The stereo configuration of the glycoside was specifically recognized by *β*-Glc. According to the catalytic characteristics of *β*-Glc, a fluorescent substrate was developed with a glucose group grafted through a *β*-*O*-glycosidic bond. 2-((6-hydroxy-2,3-dihydro-1*H*-xanthen-4-yl)methylene)malononitrile (**DXM-OH**) was chosen as an intramolecular charge transfer (ICT) fluorophore which exhibited good photostability and excellent biocompatibility (Liu, et al., 2018; Pang, et al., 2020; Sun, et al., 2019). Therefore, using **DXM-OH**, a novel fluorescent probe (**DXM-Glc**) was developed for sensing of *β*-Glc (Figure 1A). Using HPLC analysis, the hydrolysis of **DXM-Glc** mediated by *β*-Glc was confirmed and the production of **DXM-OH** was observed (Supplementary Figure S10). Herein, the absorption spectra of both **DXM-Glc** and **DXM-OH** have been measured, and a red-shift was observed for **DXM-OH** in comparison with **DXM-Glc** (Figure 1B). **DXM-Glc** was non fluorescent, due to reduced intramolecular charge transfer (ICT), while **DXM-OH** exhibited strong fluorescence, which could be used detect *β*-Glc sensitively and without interference from biological samples (Figure 1C).

**FIGURE 2:**
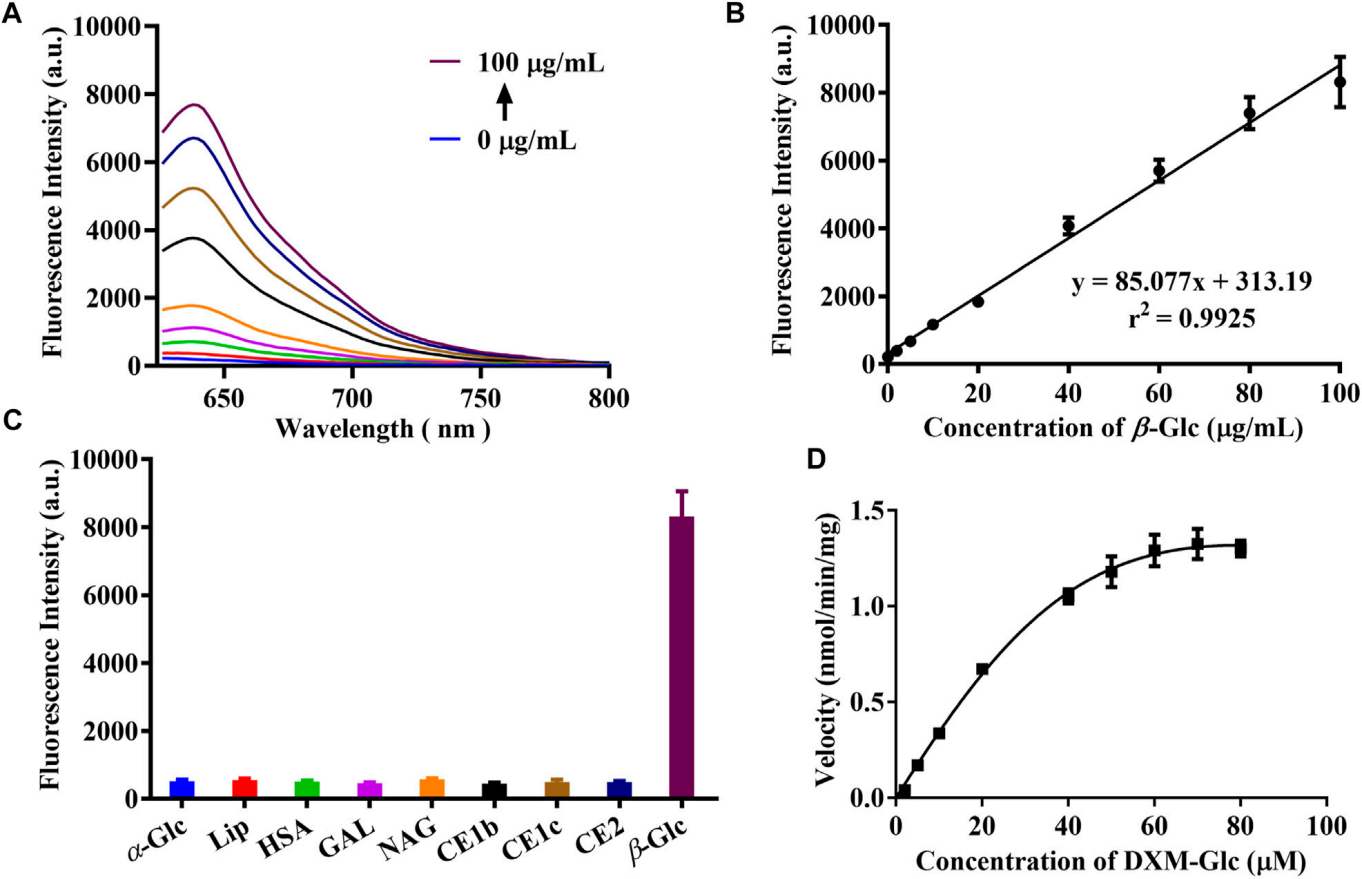
**(A)** The fluorescence response of **DXM-Glc** toward *β*-Glc with different concentrations (0–100 μg/ml). **(B)** The linear relationship between the fluorescence intensity and *β*-Glc concentrations. **(C)** The fluorescence response of **DXM-Glc** toward different enzymes. **(D)** The hydrolysis kinetics of **DXM-Glc** mediated by *β*-Glc.

For the development of a fluorescent probe for *β*-Glc, various validations were performed for the enzymatic reaction between **DXM-Glc** and *β*-Glc. Firstly, a series of fluorescence spectra were acquired for the enzymatic hydrolysis of **DXM-Glc** with different amounts of *β*-Glc (Figure 2A). The fluorescence intensity and *β*-Glc concentrations exhibited a good linear relationship (Figure 2B), which indicated the potential application for accurate determination of *β*-Glc activity. The limit of detection (LOD) was calculated to be 0.02 mU/mL (0.2 μg/ml) using 3*σ*/slope, indicating excellent sensitivity of **DXM-Glc** toward *β*-Glc. As a designed fluorescent probe for *β*-Glc, the substrate specificity of **DXM-Glc** was evaluated in presence of various species. Common ions and amino acids exhibited no interference for the fluorescence intensity of **DXM-Glc**, indicating the suitability of the system for application for the assay of biological samples (Supplementary Figure S12). More importantly, **DXM-Glc** exhibited good selectivity toward *β*-Glc in comparison with various biological enzymes, especial other glycosidases, such as *α*-Glc, GAL and NAG (Figure 2C). Thus, **DXM-Glc** was determined to be a reliable substrate for *β*-Glc. In addition, the enzymatic hydrolysis of **DXM-Glc** mediated by *β*-Glc exhibited Michaelis-Menten kinetics, with *K*_m_ = 45.97 μM and *V*_max_ = 2.19 nmol/min/mg. Therefore, **DXM-Glc** was a highly sensitive and selective fluorescent probe for *β*-Glc in a biological environment.

### Visual Identification of *β*-Glc Activity in Intestinal Fungi

**FIGURE 3:**
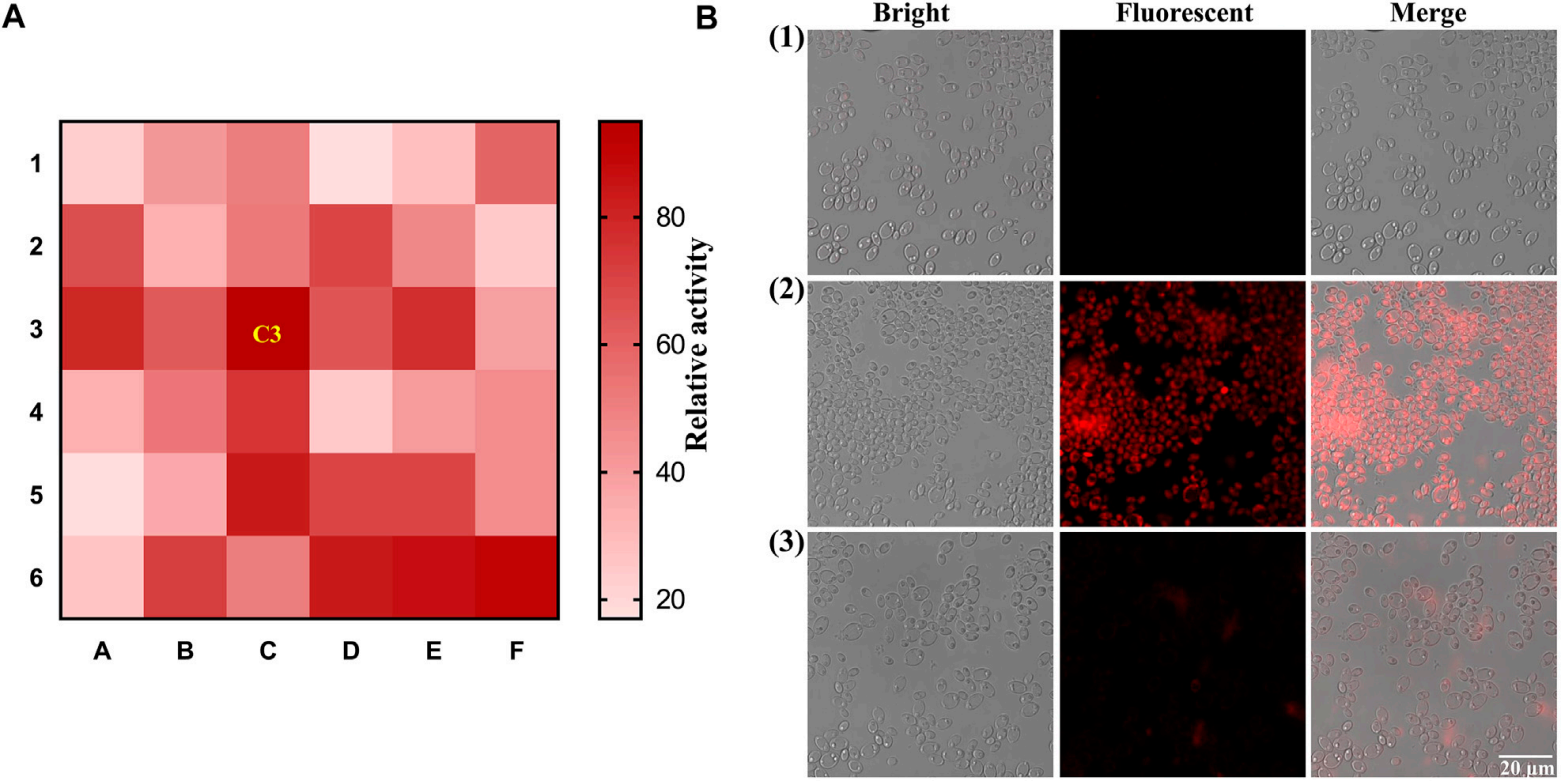
Visual identification of intestinal fungi with strong activity of *β*-Glc. **(A)** The heat-map for the screening of intestinal fungal *β*-Glc. **(B)** Fluorescence imaging of *Pichia terricola* M2 stained by **DXM-Glc** using Confocal laser scanning microscope. (1) Blank group. (2) *Pichia terricola* M2 stained by **DXM-Glc**. (3) *Pichia terricola* M2 stained by **DXM-Glc** in the presence of miglitol.

As mentioned above, intestinal bacteria are involved in the metabolism of glycosides. However, limited research on the *β*-Glc of intestinal fungi has been reported. Herein, we identified *β*-Glc active fungi using the developed fluorescent probe. 36 intestinal fungi strains were cultured with **DXM-Glc** and the fluorescence intensity of fungal culture medium was measured, so as to determine the *β*-Glc activity. As shown in Figure 3A, the C3 position of the heat map indicated the strongest fluorescence intensity along with the *β*-Glc, which corresponded to the intestinal fungus *Pichia terricola* M2. Although it has been reported that *β*-Glc produced by Pichia genus (Zhang, et al., 2020), this research represents the first exploration of *β*-Glc in *Pichia terricola* species. In order to monitor intracellular *β*-Glc, we evaluated the activity visually using the fluorescence imaging of *Pichia terricola* M2 stained using **DXM-Glc**. Compared with the blank group, strong fluorescence was observed for *Pichia terricola* M2 cells (Figure 3B). In addition, when the *β*-Glc inhibitor miglitol was co-incubated with **DXM-Glc** (Zamoner, et al., 2019), the *Pichia terricola* M2 cells displayed weaker fluorescence, indicating that the fluorescence sensing of *Pichia terricola* M2 by **DXM-Glc** was *β*-Glc dependent. Therefore, the fluorescent probe **DXM-Glc** could be used to visually identify intestinal fungus exhibiting *β*-Glc activity. Based on the excellent cell permeability, **DXM-Glc** could be used as a practical tool for imaging endogenous *β*-Glc in living fungal cells. In addition, the identified intestinal fungus *Pichia terricola* M2 is an important resource for investigating the metabolism of glycosides in the gut.

### Metabolism of Icariin by Intestinal Fungus *Pichia terricola* M2

**FIGURE 4:**
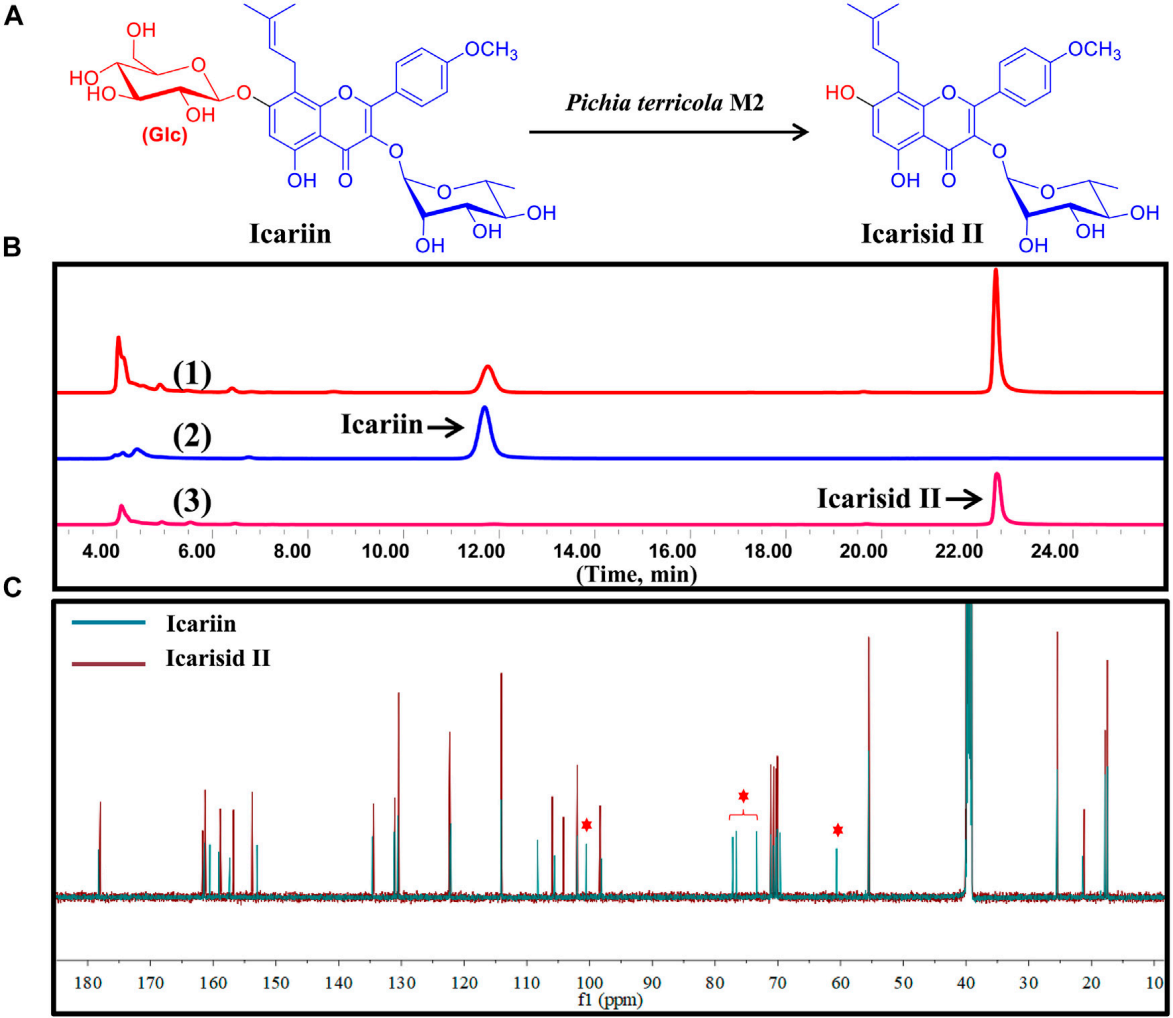
Icariin was transformed to Icarisid Ⅱ by intestinal fungus *Pichia terricola* M2. **(A)** Illustration for the biotransformation of Icariin by *Pichia terricola* M2. **(B)** HPLC chromatograms for the analysis of biotransformation of Icariin by *Pichia terricola* M2. (1) Co-incubation of Icariin and *Pichia terricola* M2. (2) Icariin reference. (3) Icarisid Ⅱ reference. **(C)** Comparison of the ^13^C NMR spectra of Icariin and Icarisid Ⅱ.

Icariin (ICA), a natural flavonoid glycoside containing glucose and rhamnose, is the major constituent of herba Epimedii (>0.05%). ICA exhibits various biological activities, including anti-inflammatory, antidepressant, antioxidative, antiatherosclerosis, anticancer, and insulin resistance(Sun, et al., 2020). Icariside II (ICA II, Baohuoside I) is one of the metabolites of ICA, which is a loss of the glucosyl moiety at the C-7 position of ICA (Figure 4A). Recently, metabolic and pharmacokinetic studies have revealed that ICA is metabolized by intestinal microbiota *in vivo* and absorbed as ICA II(Cheng, et al., 2016). Though both ICA and ICA II exhibited many common pharmacological effects, ICA II exhibits stronger biological activity when compared with ICA. Therefore, it was proposed that ICA II is the activated form of ICA *in vivo*.

In the present study, *Pichia terricola* M2 could catalyze the cleavage of the *β*-*O*-glycoside bond between glucose and flavone, producing ICA II as the metabolite (Figure 4A). This special enzymatic biotransformation of ICA was confirmed using HPLC analysis, where ICA II was found as the sole chromatographic peak (Figure 4B). Through a comparison of the ^13^C NMR spectra of ICA and ICA II confirmed removal of the glucose moiety (*δ*_C_ 60–105 ppm) (Figure 4C), which indicated the substrate specificity of *β*-Glc in *Pichia terricola* M2. Therefore, *Pichia terricola* M2 an intestinal fungus with *β*-Glc activity could transform ICA to ICA II efficiently, which not only indicated the intestinal metabolism of ICA *in vivo*, but also could be used to prepare ICA II as a bioactive substance for further pharmaceutical development.

### Icarisid Ⅱ as a Cytotoxic Agent Against Human Endometrial *Cancer* Cells

**FIGURE 5:**
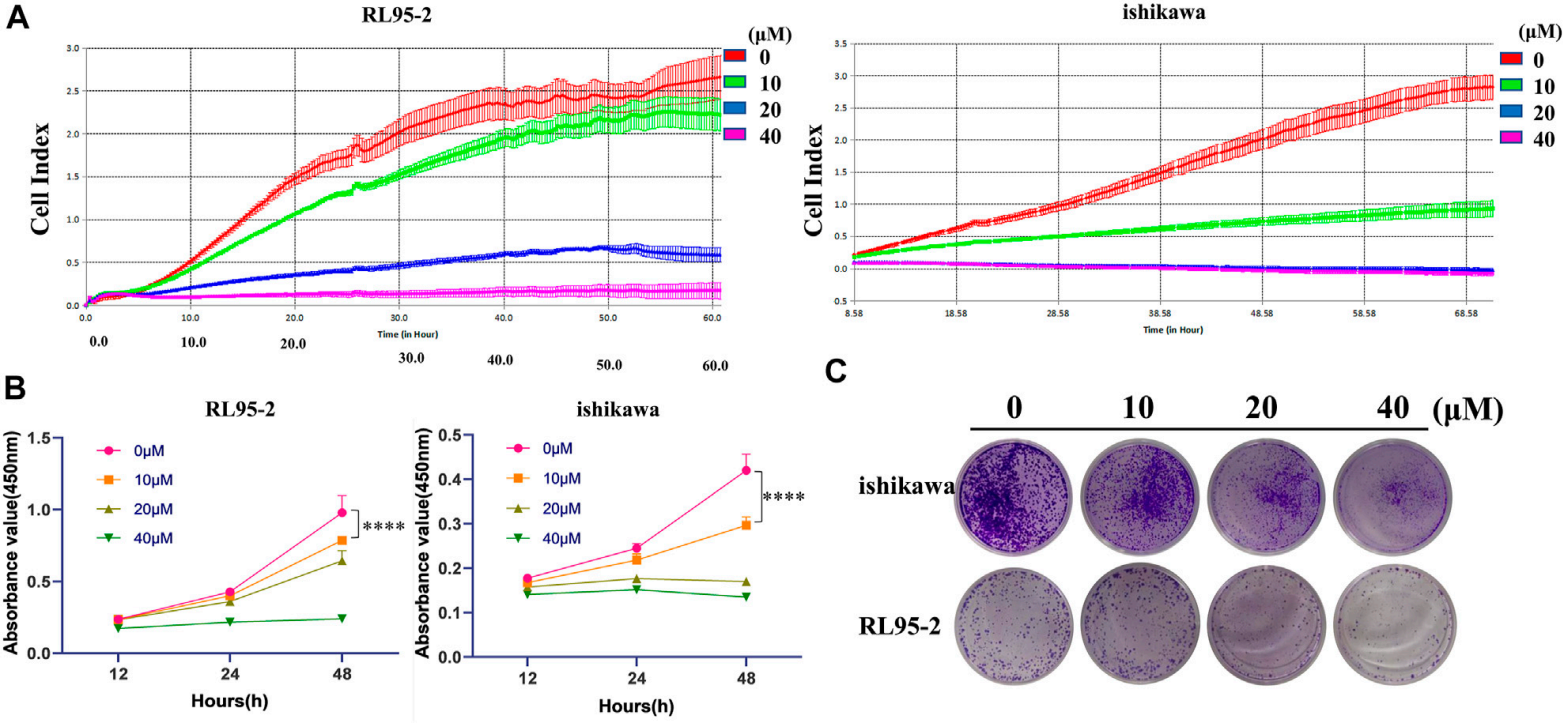
Icarisid Ⅱ displayed cytotoxic effect against human endometrial cancer cells. **(A)** Grow curves of cancer cells RL 95-2 and ishikawa in the presence of Icarisid Ⅱ. **(B)** Cell viability of RL 95-2 and ishikawa in the presence of Icarisid Ⅱ at 12, 24, and 48 h. **(C)** Inhibitory effect of Icarisid Ⅱ against cell colony formation of RL 95-2 and ishikawa.

As mentioned above, ICA Ⅱ is a metabolite of ICA mediated by *Pichia terricola* M2. It was proposed that ICA Ⅱ would exhibit improved membrane permeability enhancing bioavailability. As such, we evaluated the cytotoxicity of ICA Ⅱ toward two endometrial cancer cell lines. Therefore, we monitored the growth curves of RL95-2 and ishikawa for >60 h, to determine the effect of ICA Ⅱ on cancer cells. From these experiments it was clear that ICA Ⅱ could inhibit the growth of RL95-2 at 20 μM, and interfere with the growth of ishikawa at 10 μM (Figure 5A). Using a CCK8 assay for cancer cell viability, ICA Ⅱ also exhibited cytotoxicity against both RL95-2 and ishikawa cancer cells (Figure 5B). Furthermore, the biological effect of ICA Ⅱ on cell colony formation was evaluated in addition to the proliferation inhibitory effect. ICA Ⅱ significantly suppressed colony formation in a concentration dependent manner (Figure 5C). So, ICA Ⅱ as a metabolite of ICA exhibited significant cytotoxicity towards human endometrial cancer cells, which could be generated using intestinal fungus. These results confirm the metabolism of ICA in the gut by intestinal microbiota generating ICA Ⅱ, which could indicate a key biological function of ICA.

## Conclusions

*β*-Glc is an enzyme able to hydrolyze the *β*-glycosidic bond, and has been used as a biocatalyst in the industrial preparation of target materials. In addition, it is found in intestinal microbiota where it is involved in the metabolism of glycosides in foods or pharmaceutical substances, enhancing the absorption, bioavailability, and bioactivity of the glycosides. In this study, the conjugate of glucose and a fluorophore linked through a *β*-*O*-glycosidic bond has been developed as an enzymatic activatable fluorescent probe (**DXM-Glc**) for *β*-Glc. **DXM-Glc** exhibited high selectivity and sensitivity toward *β*-Glc. Using **DXM-Glc**, *Pichia terricola* M2 was identified as intestinal fungus expressing *β*-Glc, which could transform ICA to ICA Ⅱ efficiently. Furthermore, ICA Ⅱ was found to significantly inhibit the proliferation of human endometrial cancer cells. Therefore, the **DXM-Glc** probe can be used to evaluate the *β*-Glc activity of intestinal microbiota and monitor the metabolism of bioactive substances.

## Data Availability

The original contributions presented in the study are included in the article/Supplementary Material, further inquiries can be directed to the corresponding authors.
