# Supplementary material for: Visual Sensing of β-Glucosidase From Intestinal Fungus in the Generation of Cytotoxic Icarisid II
Source: Front Chem. 2022 May 27;10:919624. doi: 10.3389/fchem.2022.919624 (PMC9184716; doi:10.3389/fchem.2022.919624)
Supplement: Supplementary file 1 [file DataSheet1.docx]

**Supplementary Materials**

**Visual Sensing of *β*-Glucosidase in Intestinal Fungus for The Preparation of Cytotoxic Icarisid Ⅱ**

***Gang Wang ^1,†^, Fei Yan ^1,2†^, Yufei Wang ^1^, Yingping Liu ^1^, Jingnan Cui ^3^, Zhenlong Yu ^1^, Lei Feng ^2^,*** ***Tony D. James ^4,5^*, Chao Wang ^1^*, Ying Kong^1,^****

*^1^ College of Basic Medical Sciences, College of Pharmacy, Academy of Integrative Medicine, Dalian Medical University, Dalian 116044, P.R. China*

*^2^ Second Affiliated Hospital of Dalian Medical University, Dalian 116023, P.R. China*

*^3^ State Key Laboratory of Fine Chemicals, Dalian University of Technology, Dalian 116024, P.R. China*

*^4^* *Department of Chemistry, University of Bath, Bath BA2 7AY, UK*

*^5^ School of Chemistry and Chemical Engineering, Henan Normal University, Xinxiang 453007, P.R. China*

*** Correspondence:**Tony D. James

t.d.james@bath.ac.uk

Chao Wang

wach_edu@sina.com (C. Wang)

Ying Kong

yingkong@dmu.edu.cn (Y. Kong)

^†^ G. Wang and F. Yan contributed equally to this work.

Index

[Scheme S1. The synthetic route of **DXM-Glc**. S3](#_Toc94169137)

[Figure S1. ^1^H NMR (600 MHZ, DMSO-*d*_6_) of **DXM-Glc**. S3](#_Toc94169138)

[Figure S2. ^13^C NMR (150 MHZ, DMSO-*d*_6_) of **DXM-Glc**. S4](#_Toc94169139)

[Figure S3. HRMS of **DXM-Glc**. S4](#_Toc94169140)

[Figure S4. ^1^H NMR (600 MHz, DMSO-*d*_6_) spectrum of Icariin. S5](#_Toc94169141)

[Figure S5. ^13^C NMR (150 MHz, DMSO-*d*_6_) spectrum of Icariin. S5](#_Toc94169142)

[Figure S6. HR-MS of Icariin. S6](#_Toc94169143)

[Figure S7. ^1^H NMR (600 MHz, DMSO-*d*_6_) spectrum of Icarisid II. S6](#_Toc94169144)

[Figure S8. ^13^C NMR (150 MHz, DMSO-*d*_6_) spectrum of Icarisid II. S7](#_Toc94169145)

[Figure S9. HR-MS of Icarisid II. S7](#_Toc94169146)

[Figure S10. HPLC chromatograms for the hydrolysis of **DXM-Glc** mediated by *β*-GLC. (1) Control group of *β*-GLC. (2) Co-incubation of **DXM-Glc** and *β*-GLC. (3) **DXM-OH** reference. (4) **DXM-Glc** reference. S8](#_Toc94169147)

[Figure S11. The fluorescence intensity of **DXM-Glc** and **DXM-OH** in phosphate buffered solutions with different pH values. S8](#_Toc94169148)

[Figure S12. The fluorescence responses of **DXM-Glc** toward various species. S9](#_Toc94169149)

Scheme S1. The synthetic route of **DXM-Glc**.


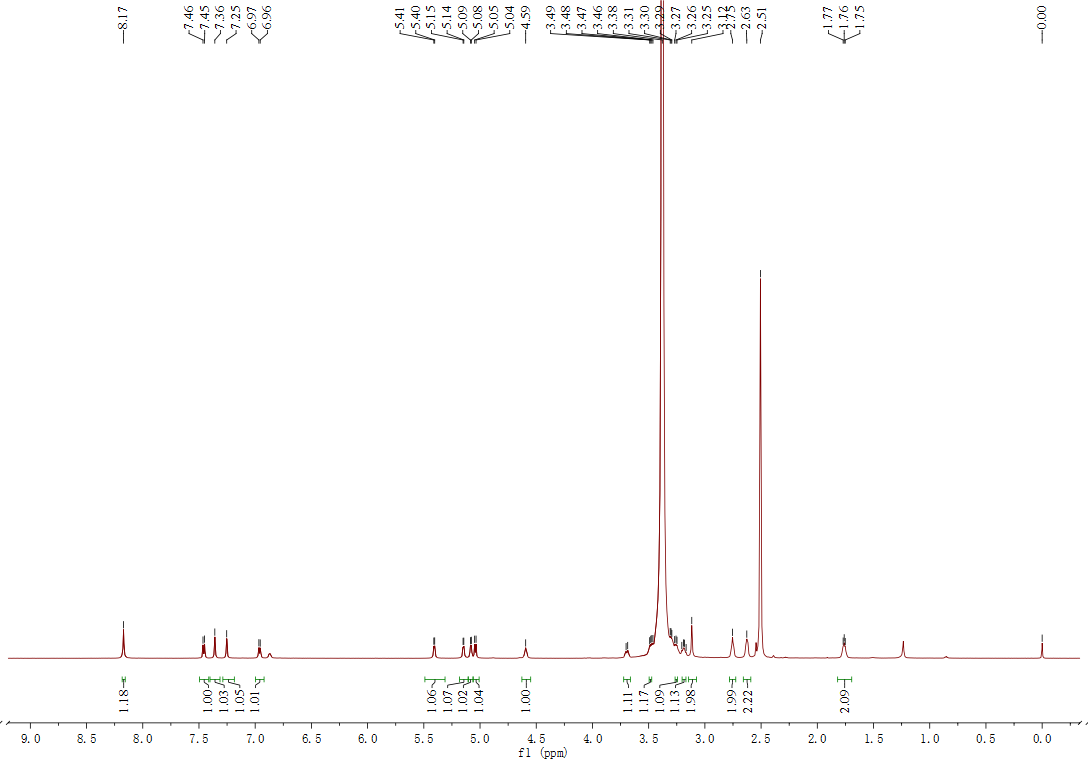


Figure S1. ^1^H NMR (600 MHZ, DMSO-*d*_6_) of **DXM-Glc**.


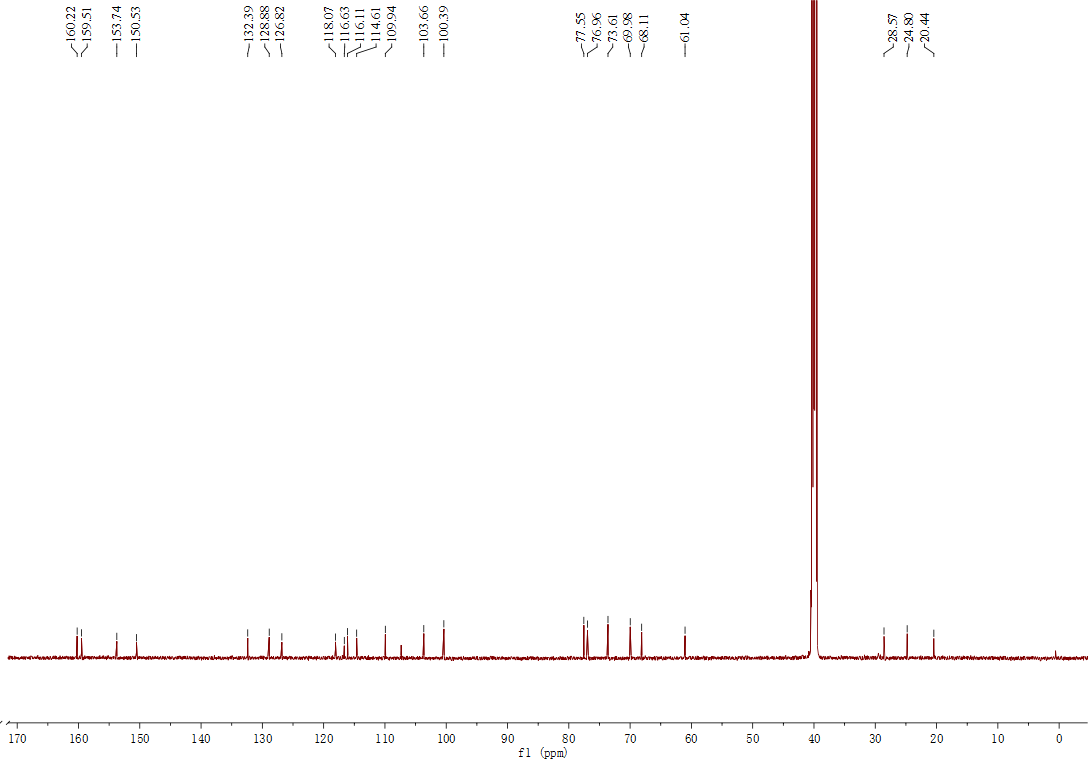


Figure S2. ^13^C NMR (150 MHZ, DMSO-*d*_6_) of **DXM-Glc**.

Figure S3. HRMS of **DXM-Glc**.


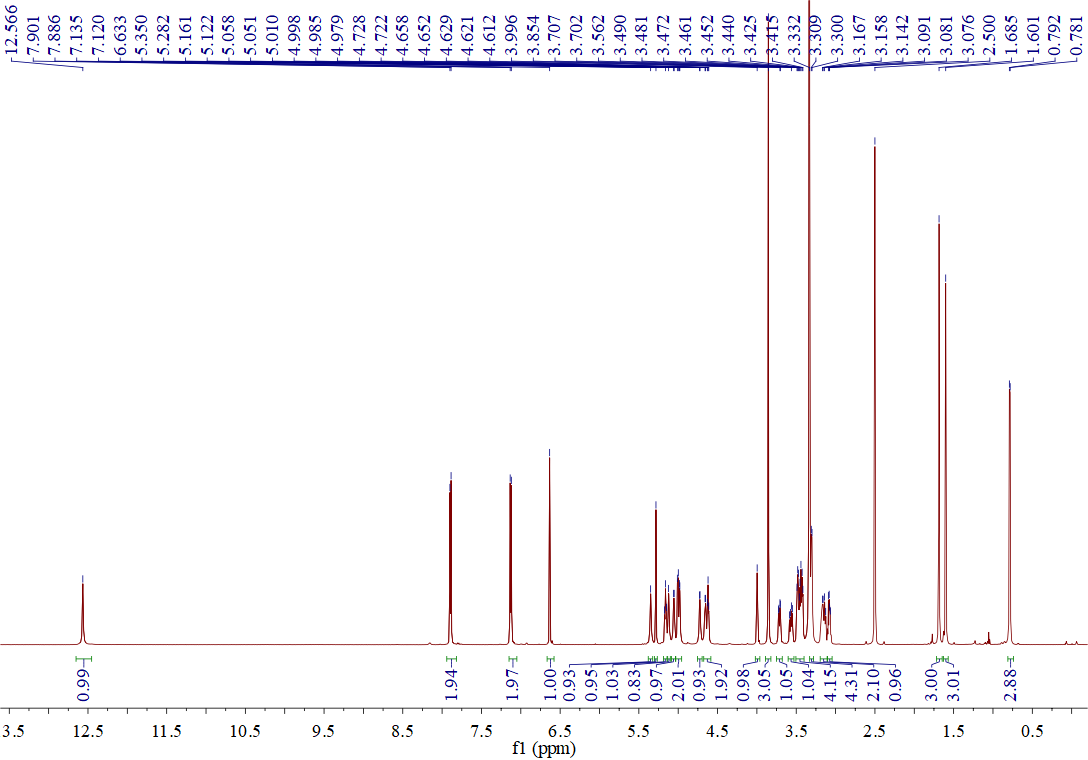


Figure S4. ^1^H NMR (600 MHz, DMSO-*d*_6_) spectrum of Icariin.


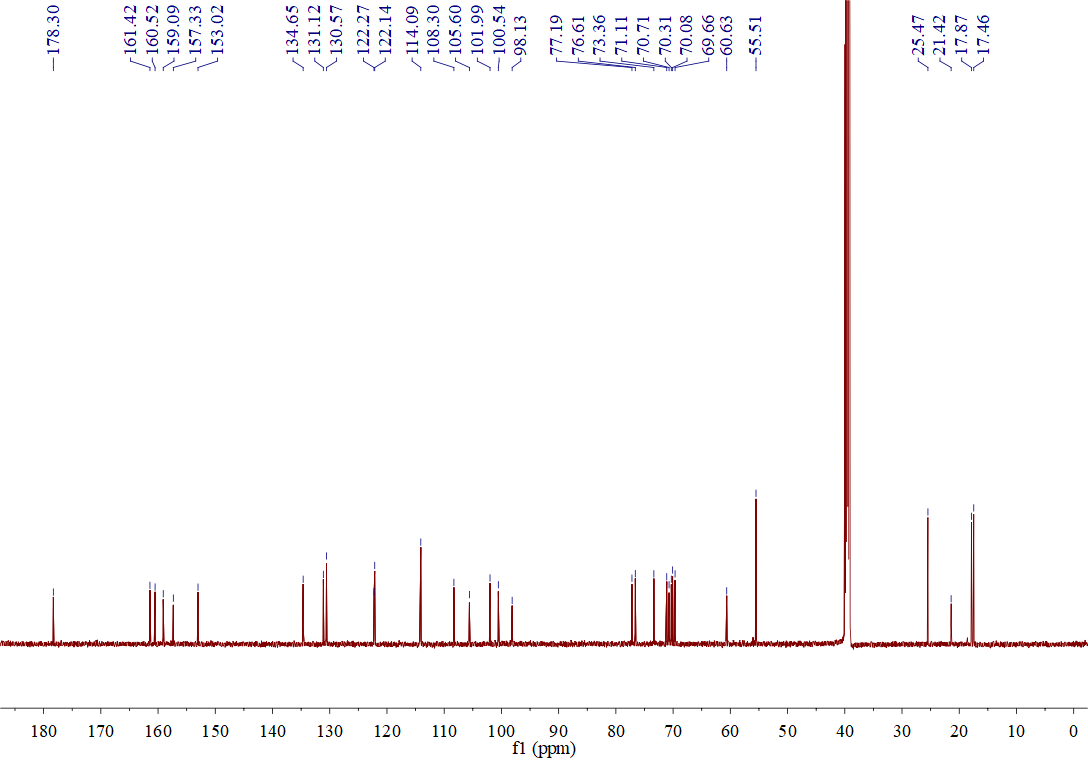


Figure S5. ^13^C NMR (150 MHz, DMSO-*d*_6_) spectrum of Icariin.

Figure S6. HR-MS of Icariin.


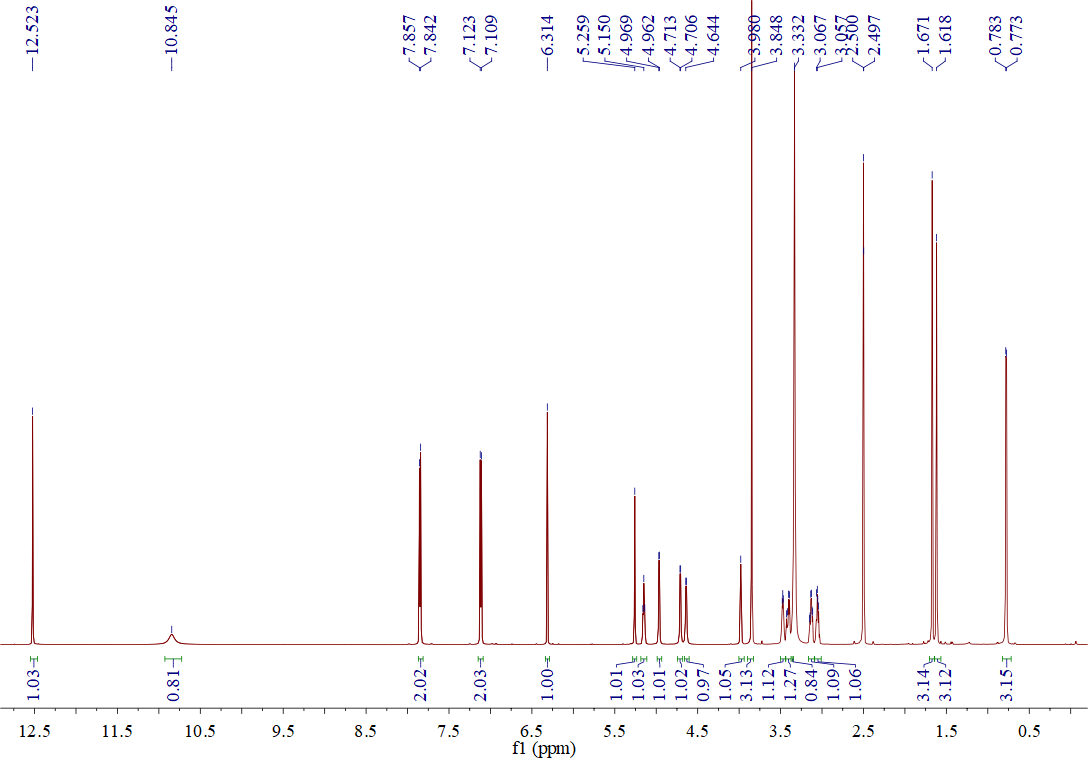


Figure S7. ^1^H NMR (600 MHz, DMSO-*d*_6_) spectrum of Icarisid II.


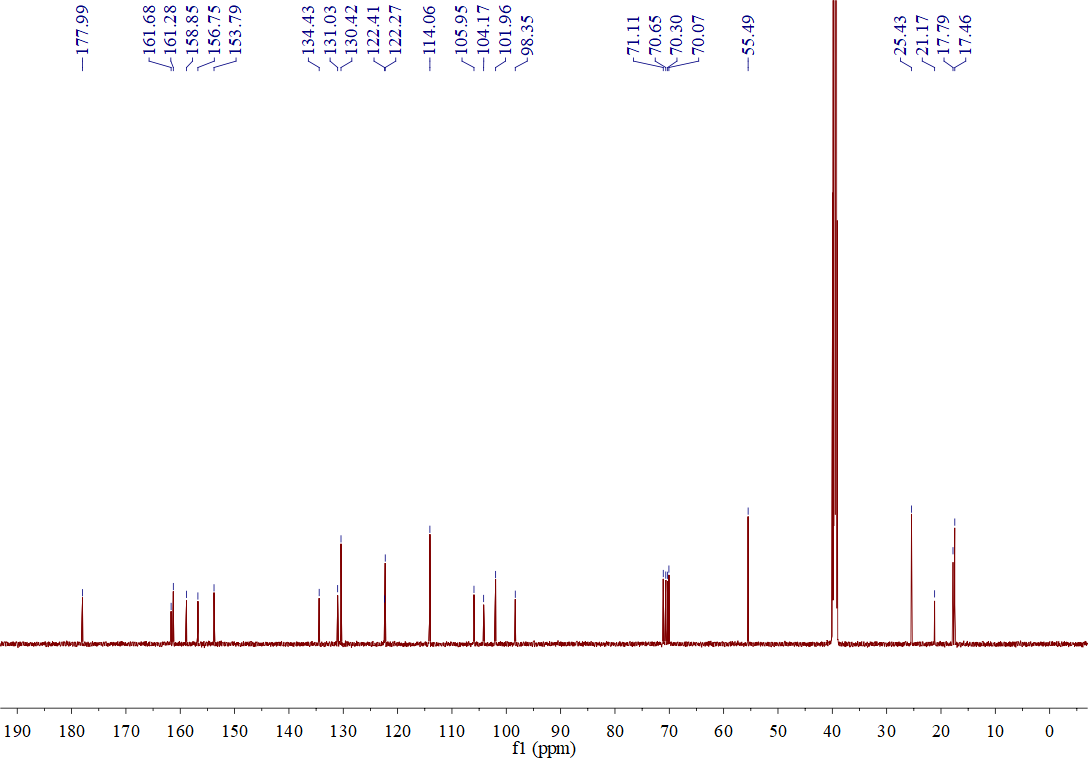


Figure S8. ^13^C NMR (150 MHz, DMSO-*d*_6_) spectrum of Icarisid II.

Figure S9. HR-MS of Icarisid II.


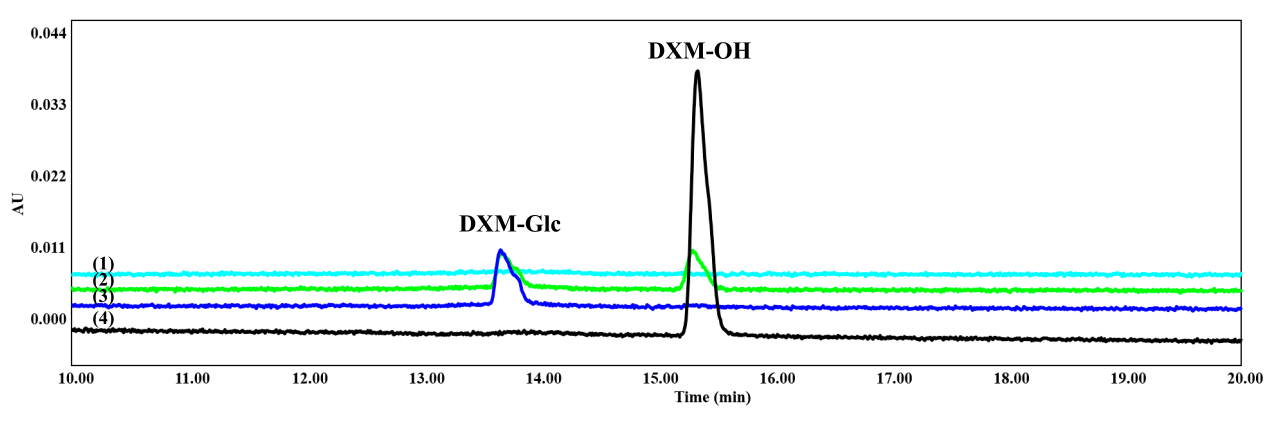


Figure S10. HPLC chromatograms for the hydrolysis of **DXM-Glc** mediated by *β*-Glc. (1) Control group of *β*-Glc. (2) Co-incubation of **DXM-Glc** and *β*-Glc. (3) **DXM-Glc** reference. (4) **DXM-OH** reference.


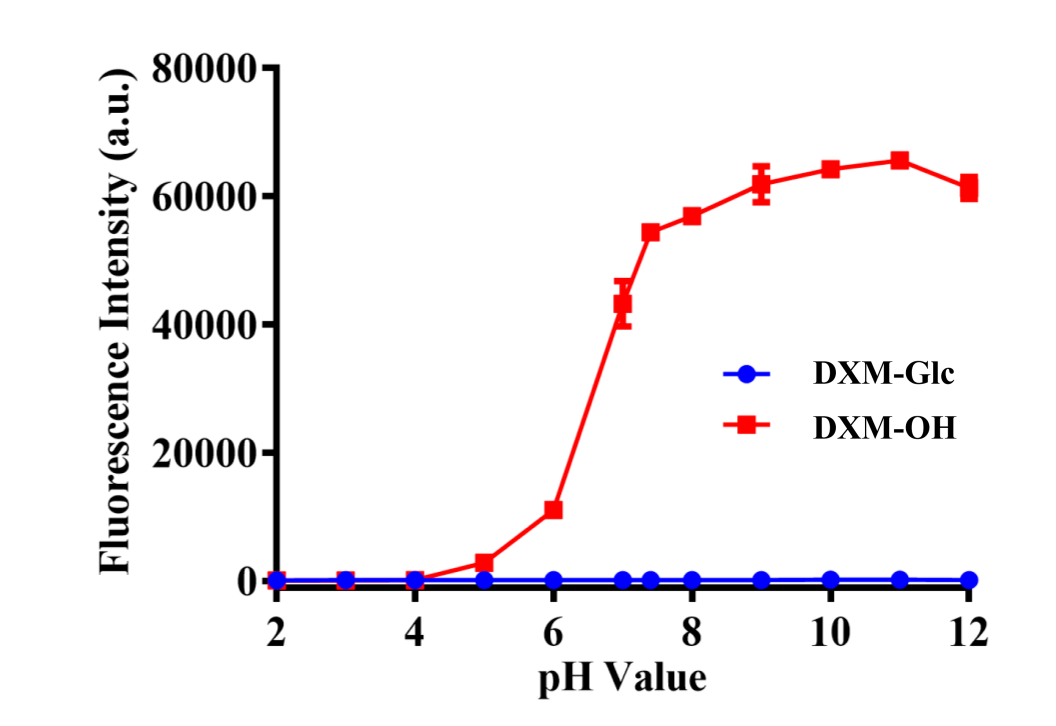


Figure S11. The fluorescence intensity of **DXM-Glc** and **DXM-OH** in phosphate buffered solutions with different pH values.


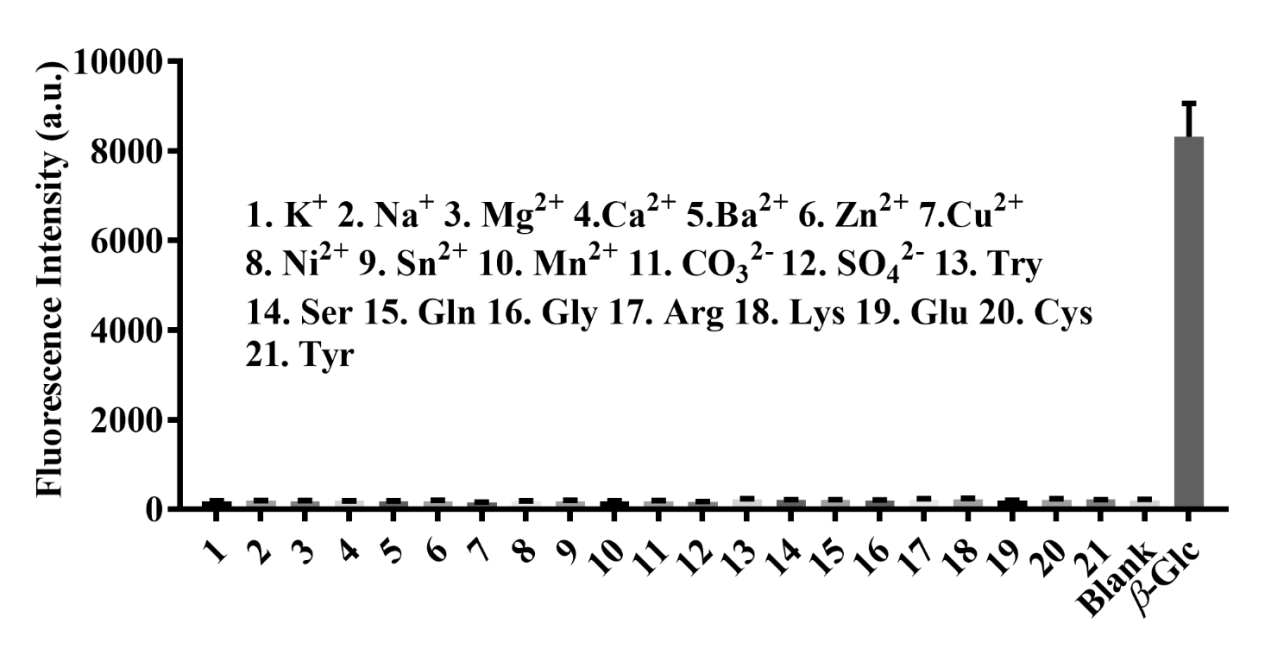


Figure S12. The fluorescence responses of **DXM-Glc** toward various species.
